# Administration of Anæsthetics

**Published:** 1895-11-02

**Authors:** Benjamin Ward Richardson


### ADMINISTRATION OF ANÆSTHETICS.

By Sir Benjamin Ward Richardson, M.D., F.R.S.

In four previous papers I have given an account of the causes of the large number of fatalities from chloroform, and I regret to say that every day seems, practically, to confirm my opinion that deaths from chloroform administration as well as from other anæsthetics seem largely on the increase. To say nothing of the deaths that are not reported, it would appear that during the past year there have been no fewer than 48 recorded cases, death in the majority taking place before any operation was performed. In 42 examples of these 48 the deaths were from pure chloroform, and out of the total number of deaths 37 took place before any operation was commenced. Of the deaths not attributable to chloroform purely one was from what is called the A. E. C. mixture, three were from mixtures of chloroform and ether, two were from ether, and one was from nitrous oxide gas. The whole means nearly one death per week, which is a sad number, and will lead before long to wider public inquiry. It is certainly far beyond anything that has occurred before, and there must be a reason for it.

In early days we considered that death by chloroform was probably due to rapidity of administration. Snow never varied from that view; Simpson did; and so there was produced a kind of fixed battle on the whole question. Snow, on his side, held to the fact that he had never had a death in his practice, although he had administered to over 4,000 patients. He thought the reason of his success was perfectly clear, and that when death did take place in the administrations of other men the subject of it must have been in a diseased condition. I was, at first, rather inclined to think that he carried his argument too far, because in two large hospitals, where no apparatus was used, and where the quantity of chloroform administered was free and rapid, I reckoned up 17,000 administrations without a death. It seemed, therefore, to me, at one time, that there might have been some other cause at work, when death followed, than rapidity of administration. Afterwards however, it appeared that, in the main, Snow was right and that the large increase of deaths which has occurred since his time has been due to the attempts, practically, to oppose his theory. I was much struck, in this direction, by a letter published on November 23rd. 1892, by Dr. Robert Bell, the senior physician to the Glasgow Hospital for Women, on the chloroform question. Bell quotes the experience of Mr. William Martin Coates, who expressed his conviction, in 1858, that chloroform could only be safely administered by limiting the dose to the smallest quantity capable of inducing insensibility to pain. By repeated trials he found that, by means of Snow's inhaler, five minims of this anæsthetic, followed by ten in twenty seconds, fifteen in forty seconds, and fifteen every minute until the patient became insensible, and afterwards an occasional ten minims, sufficed in almost every case to produce and maintain complete anæsthesia. Very rarely twenty minims were required. In the *Lancet* of December 23rd, 1882—twenty-four years later—Mr. Coates adds that, although during twenty-four years he was never prevented from administering chloroform by extreme age or infancy, by chronically diseased heart, lungs, or kidneys, he had not had a death from chloroform. He never refused chloroform to any patient in whose case pain was anticipated. The scares, including pressure on the chest, placing the head below the level of the body, did not occur.

Insisting on the necessity of giving chloroform in small and gradually increasing doses, Coates said he was certain that some, and not a few, persons were dangerously affected by the usual doses of chloroform. In support of which he gave the following illustrations. One young woman, twenty-four years of age, was completely narcotised by five minims of chloroform. A middle-aged woman was rendered insensible to pain, during an operation lasting a quarter of an hour, by seventy-five minims, and a child by ten minims. "*Had the usual doses been given to these patients their lives would have been placed in danger.*" In no case since the year 1858 had he to use galvanism, nitrate of amyl, artificial respiration, or any other mode of resuscitation. He read with painful interest the reports of deaths from chloroform, and did not come across one in which his mode of giving it had been adopted. In every case of death in which the quantity of the anæsthetic inhaled was recorded, it was much larger than that advocated by him. In later years a quicker and more daring plan, he thought, was advised and practised (the use of a towel and similar contrivances), hence, he thought, the more frequent fatal results.

It is certainly open to the administrators of chloroform that they should fall back upon the old method and administer chloroform, if they use it, or any other anæsthetic, with less rapidity than is usually employed. It must also be the duty of the surgeon not to hurry inhalation or look askance on an anæsthetist who does not rapidly perform his task.

Snow would sometimes commence inhalation in a child by administering so little as five minims at the first. He estimated that under ten years eight minims of chloroform was fully sufficient to enter the blood, and that it was quite a surprising fact what a minute quantity would suffice. He held firmly that from 4 to 5 per cent. of chloroform vapour in the air inspired was amply sufficient for the production of the deepest anæsthesia, and that more was unnecessary and dangerous. It was for this reason that he invented the "balloon"—afterwards transformed into Clover bag—in which the vapour of chloroform was never allowed to exceed 4 to 5 per cent. He considered the apparatus troublesome, however, and, being very much pressed for quicker action, invented the inhaler which bears his name, but which he quite admitted was not so perfect as the balloon, nor so satisfactory to the administrator. What we ought now to insist upon is that the wish of the surgeon should not interfere with the safety of the patient, and that it is the surgeon's duty to wait the bidding of the anæsthetist and not press him against his judgment in order to save time; knowing that whereas he himself can operate with perfect safety in a large majority of cases, the anæsthetist never administers without a certain risk, which should be reduced by every possible means at command.

